# YES-10, A Combination of Extracts from *Clematis mandshurica* RUPR. and *Erigeron annuus* (L.) PERS., Prevents Ischemic Brain Injury in A Gerbil Model of Transient Forebrain Ischemia

**DOI:** 10.3390/plants9020154

**Published:** 2020-01-26

**Authors:** Tae-Kyeong Lee, Joon Ha Park, Bora Kim, Young Eun Park, Jae-Chul Lee, Ji Hyeon Ahn, Cheol Woo Park, Yoohun Noh, Ji-Won Lee, Sung-Su Kim, Jong Dai Kim, Moo-Ho Won

**Affiliations:** 1 Department of Neurobiology, School of Medicine, Kangwon National University, Chuncheon, Gangwon 24341, Korea; tankado92@kangwon.ac.kr (T.-K.L.); bora9417@kangwon.ac.kr (B.K.); rokoko339@kangwon.ac.kr (Y.E.P.); anajclee@kangwon.ac.kr (J.-C.L.); 2 Department of Anatomy, College of Korean Medicine, Dongguk University, Gyeongju, Gyeongbuk 38066, Korea; jh-park@dongguk.ac.kr; 3 Department of Biomedical Science and Research Institute for Bioscience and Biotechnology, Hallym University, Chuncheon, Gangwon 24252, Korea; jh-ahn@hallym.ac.kr; 4 Leefarm Co., Ltd, Hongcheon, Gangwon 25117, Korea; flfhflfh2@kangwon.ac.kr; 5 Famenity Company, Gwacheon, Geyonggi 13837, Korea; yoohun.noh@famenity.com (Y.N.); jiwon.lee@famenity.com (J.-W.L.); sungsu.kim@famenity.com (S.-S.K.); 6 Division of Food Biotechnology, School of Biotechnology, Kangwon National University, Chuncheon, Gangwon 24341, Korea

**Keywords:** Ranunculaceae, Asteraceae, common carotid artery occlusion, pyramidal neurons, reactive gliosis

## Abstract

*Clematis mandshurica* RUPR. (CMR) and *Erigeron annuus* (L.) PERS. (EALP) have pharmacological effects including anti-inflammatory activity and been used in traditional medicines in Asia. However, neuroprotective effects of CMR and/or EALP extracts against brain ischemic insults have never been addressed. Thus, the aim of this study was to examine neuroprotective effects of YES-10, a combination of extracts from CMR and EALP (combination ratio, 1:1), in the hippocampus following ischemia/reperfusion in gerbils. Protection of neurons was investigated by cresyl violet staining, fluoro-jade B histofluorescence staining and immunohistochemistry for neuronal nuclei. In addition, attenuation of gliosis was studied by immunohistochemistry for astrocytic and microglial markers. Treatments with 50 or 100 mg/kg YES-10 failed to protect neurons in the hippocampus after ischemia/reperfusion injury. However, administration of 200 mg/kg YES-10 protected neurons from ischemia/reperfusion injury and attenuated reactive gliosis. These findings strongly suggest that a combination of extracts from CMR and EALP can be used as a prevention approach/drug against brain ischemic damage.

## 1. Introduction

A temporary stop of blood supply to the whole brain causes ischemia/reperfusion injury and induces selective neuronal death in specifically vulnerable regions of the brain, such as the hippocampus [1,2]. In the hippocampus, the cornu ammonis 1 (CA1) field is a field vulnerable to ischemia/reperfusion injury [3,4]. Namely, in the CA1 field, death of pyramidal neurons occurs at about 4–5 days after ischemia/reperfusion. Thus, the process of neuronal death is termed “delayed neuronal death (DND)”. In connection with DND, gliosis of astrocytes and microglia can be observed in the CA1 field after ischemia/reperfusion [5]. With occurrence of the reactive gliosis, both pro-inflammatory and anti-inflammatory responses are triggered following ischemic insults [6]. Especially, pro-inflammatory M1 microglia are predominantly increased and distributed in the hippocampal CA1 field when reactive microgliosis occurs due to cerebral ischemia [7]. In addition, a number of studies have described that enhancement of anti-inflammatory responses can prevent neuronal damage following cerebral ischemic insults [8,9,10].

Up to now, many studies with extracts from natural resources have been conducted in order to prevent ischemia/reperfusion injury following transient brain ischemia. For example, *Chrysanthemum indicum Linné* (a family of Asteraceae) extract is able to protect pyramidal neurons in the hippocampal CA1 field from transient forebrain ischemia in gerbils by its anti-inflammatory effect [8,11]. In addition, *Rosa laevigata Michx* (a family of Rosaceae) extract can attenuate infarct size induced by transient focal cerebral ischemia in rats via suppression of apoptosis and inflammation [12]. *Clematis mandshurica* RUPR. (CMR) belongs to the family of Ranunculaceae. It has been traditionally utilized in Asia because of its pharmacological activities such as antitumor, analgesic and anti-inflammatory effects [13]. Recently, a study on the pharmacological effect of CMR extract has revealed that CMR root extract possesses anti-inflammatory activity [14]. *Erigeron annuus* (L.) PERS. (EALP) belonging to Asteraceae family has also been used for oriental medicine to treat indigestion, epidemic hepatitis, enteritis, lymphadenitis and hematuria [15,16,17]. In addition, it has been reported that EALP root extract has anti-inflammatory activity [15]. 

We previously conducted a pilot experiment whether CMR or EALP displayed neuroprotective effect against cerebral ischemia, and each extract to protect pyramidal neurons in the hippocampal CA1 field following ischemic insults when each extract was pre-treated (data not shown). In this regard, there have been some reports concerned with speculations or hypotheses to explain synergistic effect of neuroprotection when extracts from two different plants, as a combination of two extracts, are administrated. For example, a combination of two crude extracts from natural resources shows neuroprotective effect against cerebral ischemia via their synergistic effect [18,19,20]. However, it is currently unclear whether CMR and EALP might have any synergistic effects when they are used together. In addition, studies on neuroprotective effects of extract complex of both CMR and EALP against cerebral ischemia are insufficient. Therefore, the aim of the current study was to investigate neuroprotective effects of CMR and EALP extracts complex named YES-10 (combination ratio, 1:1) following transient forebrain ischemia in gerbils, which are reliable experimental animals for a model of transient forebrain ischemia [21]. Furthermore, we examined effects of these extracts on the impact of astrogliosis and microgliosis in the ischemic brain. 

## 2. Results

The mongolian gerbils used in this study had been intraperitoneally pre-treated with vehicle or 50, 100 and 200 mg/kg YES-10 once a day for 7 days and the last administration was conducted at 1 h before sham or ischemia/reperfusion surgery. Thereafter, they were subjected sham or ischemia/reperfusion operation. Five days after the operation, they were sacrificed, and their brains were analyzed.

### 2.1. Cresyl Violet (CV) Positive Cells

**Figure 1:**
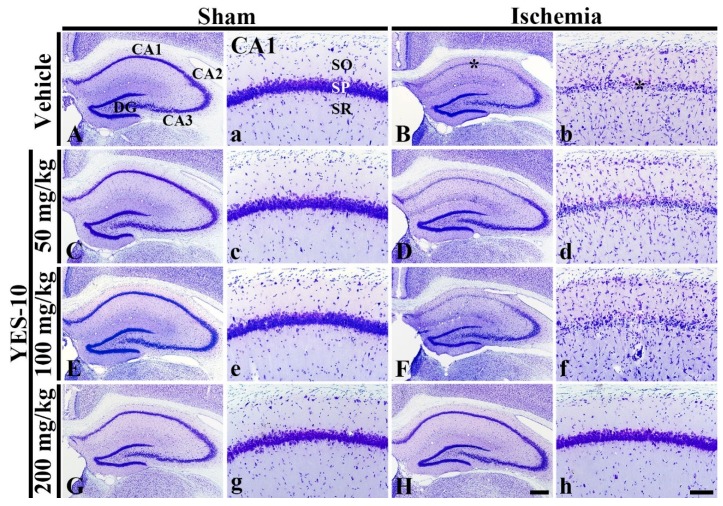
CV staining in the hippocampus (**A**–**H**) and CA1 field (**a**–**h**) of the vehicle/sham (**A**, **a**), 50 mg/kg YES-10/sham (**C**, **c**), 100 mg/kg YES-10/sham (**E**, **e**), 200 mg/kg YES-10/sham (**G**, **g**), vehicle/ischemia (**B**, **b**), 50 mg/kg YES-10/ischemia (D, d), 100 mg/kg YES-10/ischemia (**F**, **f**) and 200 mg/kg YES-10/ischemia (**H**, **h**) groups at 5 days after ischemia/reperfusion. In the vehicle/ischemia group, CV stainability is significantly decreased in the stratum pyramidal (SP) (asterisks) of the CA1 field. In the 50 and 100 mg/kg YES-10/ischemia groups, CV stainability is similar to that in the vehicle/ischemia group. However, in the YES-10/ischemia group, CV stainability is preserved. DG, dentate gyrus; SO, stratum oriens; SR, stratum radiatum. Scale bars = 400 μm (**A**–**H**) and 100 μm (**a**–**h**).

To investigate cellular morphology and distribution following ischemia/reperfusion, we performed CV staining which is used to stain Nissl substance in the cytoplasm of neurons at 5 days after sham or ischemia/reperfusion operation. CV positive cells were clearly found in all subfields of the gerbil hippocampus of the vehicle/sham group (Figure 1A,a). In the 50, 100 and 200 mg/kg YES-10/sham groups, the distribution of CV positive cells in the hippocampus was not different from that in the vehicle/sham group (Figure 1C,c,E,e,G,g). In contrast, in the vehicle/ischemia group, CV positive cells in the CA1 field, which are called CA1 pyramidal cells, were altered (Figure 1B). Namely, in this group, stainability of CV was significantly weakened in the stratum pyramidale of the CA1 field, and numbers of CV positive cells increased markedly in strata oriens and radiatum (Figure 1B,b). 

In the 50 and 100 mg/kg YES-10/ischemia groups, the distribution of CV positive cells in the CA1 field was not different compared to that in the vehicle/ischemia group. Namely, the cytoplasm of CV positive CA1 pyramidal cells was pale in CV stainability (Figure 1D,d,F,f). However, in the 200 mg/kg YES-10/ischemia group, the distribution pattern and stainability of CV positive CA1 pyramidal cells was similar to that in the vehicle/sham group (Figure 1H,h).

### 2.2. Neuronal Nuclei (NeuN) Immunoreactive Neurons

**Figure 2:**
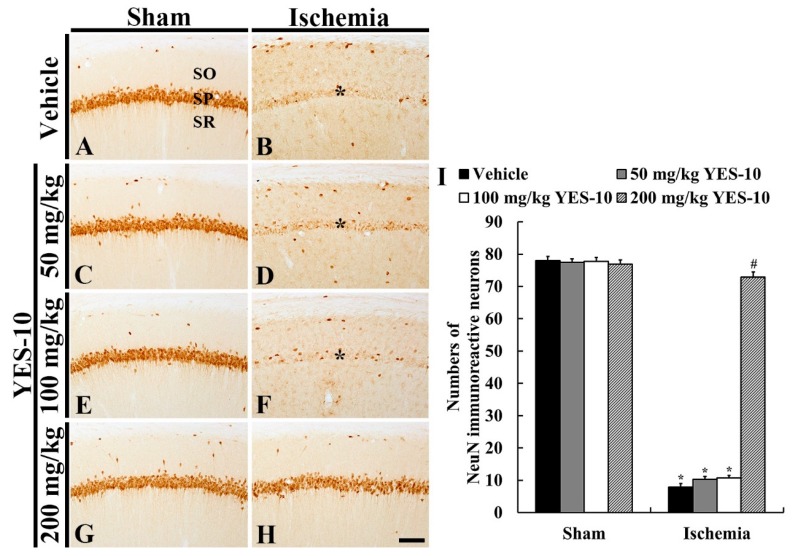
NeuN immunohistochemistry in the CA1 field of the vehicle/sham (**A**), 50 mg/kg YES-10/sham (**C**), 100 mg/kg YES-10/sham (**E**), 200 mg/kg YES-10/sham (**G**), vehicle/ischemia (**B**), 50 mg/kg YES-10/ischemia (**D**), 100 mg/kg YES-10/ischemia (**F**) and 200 mg/kg YES-10/ischemia (**H**) groups at 5 days after ischemia/reperfusion. In all sham groups, pyramidal cells in the CA1 field show strong NeuN immunoreactivity. However, most of CA1 pyramidal cells were hardly NeuN immunoreactivity in the vehicle, 50 and 100 mg/kg YES-10/ischemia groups (asterisks). In the 200 mg/kg YES-10/ischemia group, many CA1 pyramidal cells show strong NeuN immunoreactivity. Scale bar = 100 μm. (**I**) The mean number of NeuN immunoreactive cells in the CA1 field at 5 days post-ischemia (n = 7 in each group, *P < 0.05 versus vehicle/sham group, #*P* < 0.05 versus vehicle/ischemia group). The bars indicate the means ± standard error of mean (SEM) standard error mean.

To examine neuronal loss following ischemia/reperfusion, immunohistochemistry for NeuN was carried out at 5 days after sham or ischemia/reperfusion operation. In all sham groups, NeuN immunoreactive CA1 pyramidal cells were distinctly observed, and the number of NeuN immunoreactive cells in each group was not significantly different between all the sham groups (Figure 2A,C,E,G,I). In contrast, CA1 pyramidal cells hardly showed NeuN immunoreactivity in the vehicle/ischemia group, showing that a significant decrease in numbers of NeuN immunoreactive CA1 pyramidal cells was shown compared to that in the vehicle/sham group (Figure 2B,I).

In the 50 and 100 mg/kg YES-10/ischemia group, NeuN immunoreactive CA1 pyramidal cells were seldom observed, showing that numbers of NeuN immunoreactive CA pyramidal cells were not different from those in the vehicle/ischemia group (Figure 2D,F,I). On the other hand, CA1 pyramidal cells of the 200 m/kg YES-10/ischemia group showed strong NeuN immunoreactivity, and the mean number of NeuN immunoreactive CA1 pyramidal cells was significantly higher than that in the 50 and 100 mg/kg YES-10/ischemia groups (Figure 2H,I).

### 2.3. Fluoro-Jade B (F-J B) Positive Cells

**Figure 3:**
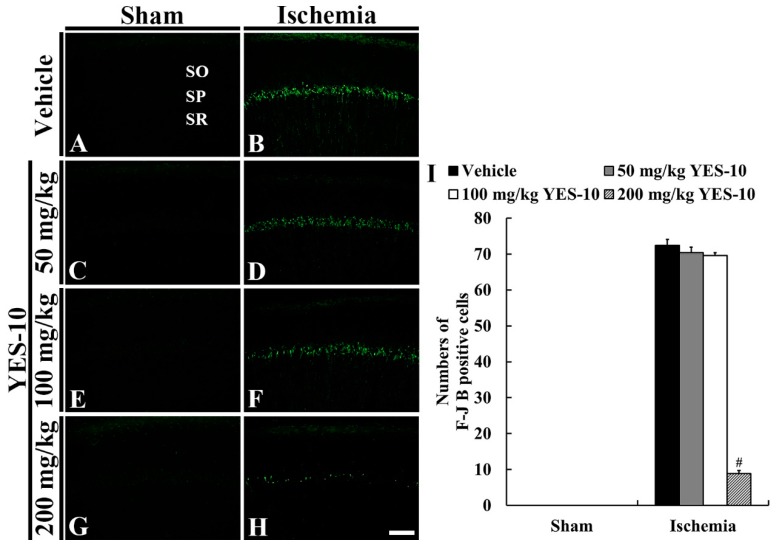
F-J B histofluorescence staining in the CA1 field of the vehicle/sham (**A**), 50 mg/kg YES-10/sham (**C**), 100 mg/kg YES-10/sham (**E**), 200 mg/kg YES-10/sham (**G**), vehicle/ischemia (**B**), 50 mg/kg YES-10/ischemia (**D**), 100 mg/kg YES-10/ischemia (**F**) and 200 mg/kg YES-10/ischemia (**H**) groups at 5 days post-ischemia. F-J B positive CA1 pyramidal cells were found in the vehicle, 50 and 100 mg/kg YES-10/ischemia groups. However, F-J B positive CA1 pyramidal cells were rarely observed in the 200 mg/kg YES-10/ischemia group. Scale bar = 100 μm. (**I**) The mean number of F-J B positive CA1 pyramidal cells at 5 days post-ischemia (n = 7 in each group, #*P* < 0.05 versus vehicle/ischemia group). The bars indicate the means ± SEM.

To detect degenerating (dead) neurons due to ischemia/reperfusion injury, we conducted F-J B, a high affinity fluorescent marker for neuronal degeneration, histofluorescence at 5 days after sham or ischemia/reperfusion operation. In all the sham groups, F-J B positive cells were not found in any layers of all the hippocampal subregions (Figure 3A,C,E,G). However, many cells in the stratum pyramidale (pyramidal cells) were positive to F-J B in the CA1 field of the vehicle/ischemia group (Figure 3B,I). 

In the 50 and 100 mg/kg YES-10/ischemia groups, F-J B positive CA1 pyramidal cells were shown, and their mean number was not significantly different from that in the vehicle/ischemia group (Figure 3D,F,I). In the 200 mg/kg YES-10/ischemia group, a few F-J B positive CA1 pyramidal cells were detected, and their mean number was significantly decreased compared to that in the vehicle/ischemia group (Figure 3H,I).

### 2.4. Glial Fibrillary Acidic Protein (GFAP) Immunoreactive Astrocytes

**Figure 4:**
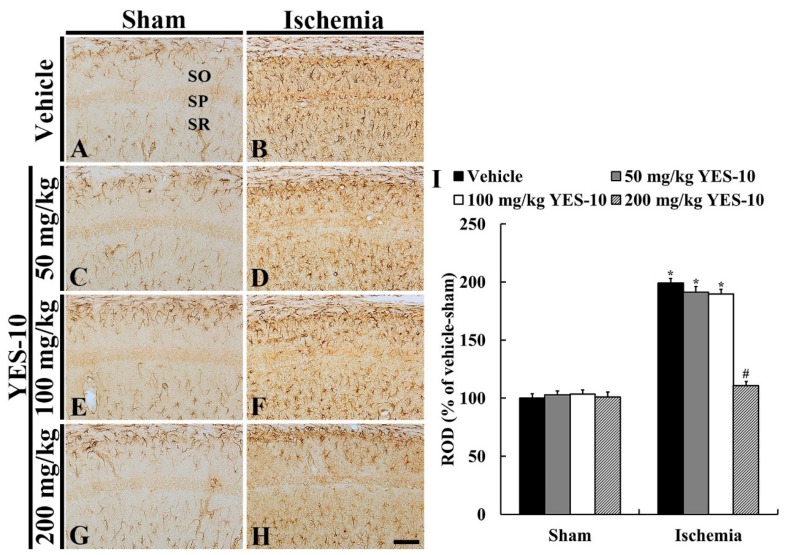
Immunohistochemistry for GFAP in the CA1 field of the vehicle/sham (**A**), 50 mg/kg YES-10/sham (**C**), 100 mg/kg YES-10/sham (**E**), 200 mg/kg YES-10/sham (**G**), vehicle/ischemia (**B**), 50 mg/kg YES-10/ischemia (**D**), 100 mg/kg YES-10/ischemia (**F**) and 200 mg/kg YES-10/ischemia (**H**) groups at 5 days post-ischemia. GFAP immunoreactive astrocytes are very similar in all the sham groups. In the vehicle/ischemia group, GFAP immunoreactive astrocytes are hypertrophied. In the 50 and 100 mg/kg YES-10/ischemia groups, GFAP immunoreactive astrocytes are similar to those in the vehicle/ischemia group. However, in the 200 mg/kg YES-10/ischemia group, the cytoplasm of GFAP immunoreactive astrocytes is lesser hypertrophied than that in the vehicle/ischemia group. Scale bar = 100 μm. (I) ROD as % of GFAP immunoreactive structures in the CA1 field at 5 days post-ischemia (n = 7 in each group, *P < 0.05 versus vehicle/sham group, #*P* < 0.05 versus vehicle/ischemia group). The bars indicate the means ± SEM.

To investigate change in astrocytes following ischemia/reperfusion, immunohistochemistry for GFAP was performed at 5 days after sham or ischemia/reperfusion operation. GFAP immunoreactive astrocytes in the CA1 field of all the sham groups were principally distributed in strata oriens and radiatum (Figure 4A). These astrocytes had a small cell body and thread-like processes (Figure 4A,C,E,G), showing that relative optical density (ROD) of GFAP immunoreactivity was similar in all the sham groups (Figure 4I). However, in the vehicle/ischemia group, GFAP immunoreactive astrocytes were altered. Namely, they had bulky cytoplasm and thickened processes (Figure 4B), showing that the ROD of GFAP immunoreactivity was significantly increased (about 198% versus vehicle/sham group) compared to that in the vehicle/sham group (Figure 4I).

In the 50 and 100 mg/kg YES-10/ischemia groups, the distribution pattern of GFAP immunoreactive astrocytes in the CA1 field was similar to that in the vehicle/ischemia group (Figure 4D,F), and their ROD was about 191% and 190%, respectively, of that in the vehicle/sham group (Figure 4I). However, in the 200 mg/kg YES-10/ischemia group, the cytoplasm of GFAP immunoreactive astrocytes was lesser hypertrophied compared to those in the other ischemia groups (Figure 4H), and their ROD was significantly lower (about 109% versus vehicle/sham group) than that in the vehicle/ischemia group (Figure 4I).

### 2.5. Ionized Calcium-Binding Adaptor Molecule 1 (Iba-1) Immunoreactive Microglia

**Figure 5:**
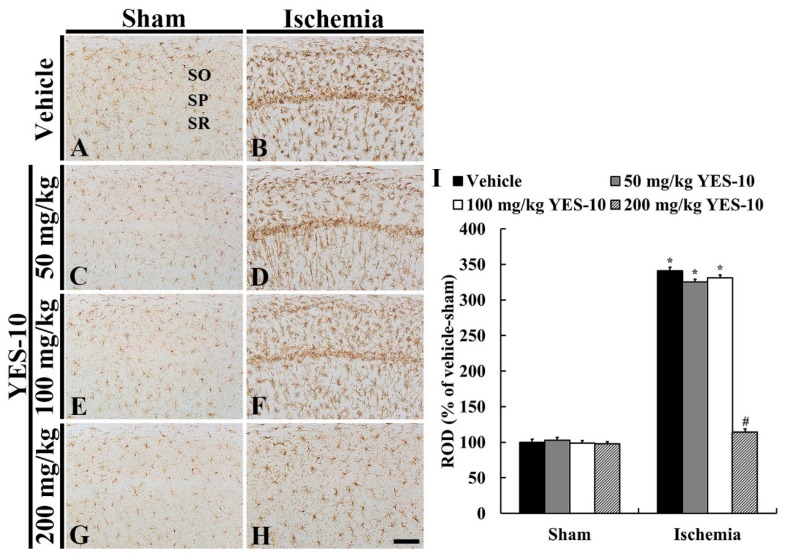
Immunohistochemistry for Iba-1 in the hippocampal CA1 field of the vehicle/sham (**A**), 50 mg/kg YES-10/sham (**C**), 100 mg/kg YES-10/sham (**E**), 200 mg/kg YES-10/sham (**G**), vehicle/ischemia (**B**), 50 mg/kg YES-10/ischemia (**D**), 100 mg/kg YES-10/ischemia (**F**) and 200 mg/kg YES-10/ischemia (**H**) groups at 5 days post-ischemia. Iba-1 immunoreactive microglia in all sham groups are similar. In the vehicle/ischemia group, the cytoplasm and processes of Iba-1 immunoreactive microglia is hypertrophied and are thickened, respectively. In the 50 and 100 mg/kg YES-10/ischemia groups, Iba-1 immunoreactive microglia are similar to those of the vehicle/ischemia group. In contrast, in the 200 mg/kg YES-10/ischemia group, Iba-1 immunoreactive microglia are less hypertrophied than those in the vehicle/ischemia group. Scale bar = 100 μm. (**I**) ROD as % of Iba-1 immunoreactive structures in the CA1 field at 5 days post-ischemia (n = 7 in each group, *P < 0.05 versus vehicle/sham group, #*P* < 0.05 versus vehicle/ischemia group). The bars indicate the means ± SEM.

To examine alteration of microglia due to ischemia/reperfusion injury, we conducted immunohistochemistry for Iba-1 at 5 days after sham or ischemia/reperfusion operation. In all the sham groups, Iba-1 immunoreactive microglia were principally scattered throughout all layers in the CA1 field (Figure 5A,C,E,G). In these groups, the microglia had a small cell body and fine processes (Figure 5A,C,E,G), showing that ROD in each group was similar (Figure 5I). In the vehicle/ischemia group, however, the cytoplasm of Iba-1 immunoreactive microglia became hypertrophied, and their processes were thickened (Figure 5B). In particular, many hypertrophied microglia converged in the stratum pyramidale (Figure 5B). In this group, the ROD of Iba-1 immunoreactivity was significantly increased (about 341% versus vehicle/sham group) compared to that in the vehicle/sham group (Figure 5I).

In the 50 and 100 mg/kg YES-10/ischemia groups, Iba-1 immunoreactive microglia were activated like those in the vehicle/ischemia group (Figure 5B,D,F), and their ROD was about 325% and 331%, respectively, of that in the vehicle/sham group (Figure 5I). However, in the 200 mg/kg YES-10/ischemia group, the cytoplasm of Iba-1 immunoreactive microglia was less hypertrophied than that in the vehicle/ischemia group (Figure 5H). In addition, the ROD of them was significantly low (about 114% versus vehicle/sham group) compared to that in the vehicle/ischemia group (Figure 5I).

## 3. Discussion

In this study, we investigated neuroprotective effect of YES-10 against ischemic insult by using histological analyses. CV staining, immunohistochemistry for NeuN and F-J B histofluorescence staining revealed neuroprotective effect when 200 mg/kg YES-10 was pre-treated. Additionally, immunohistochemistry for GFAP and Iba-1, showed that pre-administration of 200 mg/kg YES-10 significantly alleviated reactive gliosis following ischemia/reperfusion.

Generally, 100 mg/kg of single compound drugs must be considered overdose. However, dose of 100 mg/kg or 200 mg/kg YES-10, used in our current study, may be not overdose since it is crude extract obtained from natural resources. A number of previous studies on neuroprotective effects by crude extracts derived from plants against cerebral ischemic injuries have used 200 mg/kg of dose. For example, for *Chrysanthemum indicum Linné* extract [8,11], *Populus tomentiglandulosa* extract [5,22], *Oenanthe Javanica* extract [1,23] and *Glehnia littoralis* extract [24] 200 mg/kg were used. In addition, these studies showed that treatment with dose of 100 mg/kg failed to protect neurons but administration of 200 mg/kg exhibited neuroprotective effects against cerebral ischemic injury. YES-10 used in our present study is a combination of extract from CMR and EALP (combination ratio, 1:1). In this regard, we assure that 200 mg/kg YES-10 is not overdose since toxicity by treatment with 200 mg/kg YES-10 had not been shown when we examined the livers and kidneys with the naked eye (data not shown).

Studies on the application of study on the application of CMR and EALP extracts to study neuroprotective effects in animal models of transient cerebral ischemia have not been performed. When lower dose of YES-10 (50 or 100 mg/kg) were administered to gerbils before induction of ischemia/reperfusion, no neuroprotective effect was displayed. However, when 200 mg/kg YES-10 was used for pretreatment, pyramidal cells in the hippocampal CA1 field were protected from ischemia/reperfusion injury. There have been studies on synergistic effects. For example, a combination of *Ligusticum chuanxiong* and *Radix Paeoniae* which belongs to the family of Apiaceae and Paeoniaceae ameliorates ischemic brain damage following transient focal cerebral ischemia induced by middle cerebral artery occlusion in rats by anti-inflammatory and anti-apoptotic effects [18,19]. Furthermore, it has been reported that *Astragalus membranaceus* (a family of Astragalin) extract combined with *Panax notoginseng* (a family of Panax) extract plays neuroprotective effect via improving energy metabolism and inhibiting apoptosis against cerebral ischemia induced by bilateral common carotid arteries occlusion in mice [20]. In our current study, neuroprotective effect of 200 mg/kg YES-10 was confirmed in a gerbil model of 5-min transient forebrain ischemia, in which CA1 pyramid cells die following 5-min transient forebrain ischemia [3,25]. Neuroprotective effect in ischemia is prone to variety according to brain areas and/or degree of ischemic injury; therefore, it is needed to study the neuroprotective effects of YES-10 in more ischemia-resistant brain areas following severer transient ischemia. It has been reported that neurons in the hippocampal CA2/3 field or cerebral cortex is damaged in a gerbil model of 15-min transient forebrain ischemia [21]. In addition, substances showing neuroprotective effects necessarily should be able to penetrate the blood brain barrier (BBB) [26,27]. In this regard, it is important to investigate the absorption, distribution, metabolism and excretion (ADME properties), and the analysis of active ingredients of YES-10 that can cross the BBB. To the best of our knowledge, however, major active components of YES-10 are poorly known, and it is largely unknown which components can penetrate the BBB. In this study, we did not analyze the mechanisms of the neuroprotective effect of YES-10 against ischemia/reperfusion injury, which is a limitation of our present study.

2, 3, 5-Tiphenyltetrazolium chloride (TTC) staining assay is widely used for visualization of brain damage via staining mitochondrial endogenous dehydrogenase [28]. In this regard, TTC staining assay can detect infarcted lesion and penumbra following transient (at least one hour) or permanent focal ischemia induced by middle cerebral artery occlusion (MCAO) in rats [28,29]. However, a gerbil model of transient forebrain ischemia, which was used in this study, cannot develop infarcted lesion and penumbra. Therefore, TTC staining assay cannot be applied to our present study. Damage in a gerbil model of transient forebrain ischemia is different from MCAO model.

In the central nervous system (CNS), glial cells including astrocytes and microglia carry out various physiological functions. In the case of astrocytes, they form a BBB by providing cellular link between neuronal circuitry and blood vessels. Through such linkage, astrocytes provide nutrients to nervous tissues [30]. In the BBB, extracellular ion and water balance are controlled by various ion and water channels that are expressed in the astrocyte-endfeet [31]. In the case of microglia, they take charge as immunocytes in the CNS and secrete diverse kinds of inflammatory cytokines in specific states such as ischemic insults [32,33]. As immune cells, microglia can remove dead cells and tissue debris in the CNS under pathological conditions [34]. Excessive activation of both astrocytes and microglia is a phenomenon that occurs when the CNS is under diverse pathological states. This is termed “reactive gliosis” [35]. The reactive gliosis after longer ischemic duration inflicts damage, namely, the degree of reactive depends on time of ischemic duration [21]. In addition, it has been reported that, at prolonged point in time after transient cerebral ischemia (40 days post-ischemia), astrogliosis starts to get fade (this phenomenon means death of astrocytes) [36].

In the present study, we found that reactive gliosis was severe in the hippocampal CA1 field of the vehicle/ischemia group at five days after transient forebrain ischemia. Since the neuroprotective effect was shown in the 200 mg/kg YES-10/ischemia group, we investigated the effect of 200 mg/kg YES-10 on the reactive gliosis in the 200 mg/kg YES-10/ischemia group. In the vehicle/ischemia group, both astrocytes and microglia became significantly hypertrophied compared to those in the vehicle/sham group. In the case of microglia, we found that they were concentrated in the stratum pyramidale of the CA1 field, in which CA1 pyramidal cells were dead, at 5 days after ischemia/reperfusion. This phenomenon might be due to migration of microglia in order to clean dead pyramidal neurons [37]. In this study, reactive gliosis in the ischemic CA1 field was not attenuated at five days post-ischemia after pre-administration with 50 or 100 mg/kg YES-10. However, when we pre-treated 200 mg/kg YES-10, a significant attenuation of reactive gliosis in the ischemic CA1 field was shown at five days after ischemia/reperfusion. This finding indicates that a combination of CMR and EALP extracts can attenuate reactive gliosis induced by ischemic insults.

Hederagenin, a major component of CMR, is well known to attenuate inflammatory response [13]. Furthermore, triterpene saponins derived from *Clematis mandshurica* possess inhibitory activities on nitrous oxide (NO) production [38]. On the other hand, caffeic acid isolated from leaf of *Erigeron annuus* confers neuroprotective and antioxidant effects [39]. Moreover, 2, 3-deoxygenated flavonone and erigeroflavonone originating from *Erigeron annuus* have inhibitory properties against formation of advanced glycation end products (AGEs) and aldose reductase in rats, which are useful in attenuating complications of diabetes mellitus (DM) [40]. In this study, we found neuroprotective effects when we pretreated 200 mg/kg YES-10. For these findings, an in vitro study is needed, but our current study has a marked limitation. Many in vitro studies on neuroprotective effects have been carried out by using oxygen-glucose deprivation (OGD), and, in this model, cellular viability has been conducted via an MMT assay. In addition, in vitro BBB models have been developed and conducted for a variety of studies; however, none of them can completely replicate in vivo conditions [41]. Those in vitro investigations entirely display different approaches from our present in vivo examination to demonstrate neuroprotective effectiveness. 

In summary, 200 mg/kg YES-10, a combination of CMR and EALP extracts, protected pyramidal neurons in the hippocampal CA1 field from ischemia/reperfusion injury and attenuated reactive gliosis in the ischemic field. These findings strongly suggest that 200 mg/kg YES-10 can be a useful candidate for developing prevention against cerebral ischemic insults. Further studies are needed to study mechanisms involved in their protective effects against ischemia/reperfusion injury.

## 4. Materials and Methods

### 4.1. Preparation of CMR and EALP Extracts

CMR and EALP were cultivated with a slight reference to a precedent study [42]. In brief, they were cultivated in Metro-Mix potting soil with 5 g of Osmocote Plus slow release fertilizer (Scotts). Namely, they were grown from seeds in pots which are 17 cm diameters at 25 ± 0.5 °C and bottom-watered once a day with a hose. Growth period was 6 weeks, and they were harvested. Both CMR and EALP were washed, followed with drying to a constant weight at 50 °C and ground into powder (<1 mm). In turn, 150 g of CMR was extracted with seven-fold the volume of 50% EtOH for 60 min and refluxed two times (2 h/reflux). The resulting suspension was filtered, concentrated to be powder by using a rotary evaporator and stored at 4 °C. By using the same procedure, 150 g EALP was extracted with 50% EtOH, filtered and dried to be powder. CMR and EALP were mixed 1:1 ratio to be YES-10 which was gifted from Famenity Co., Ltd. (Yongin, Korea).

### 4.2. Experimental Animals

In total, 56 male Mongolian gerbils (6 months old and 70–80 g of body weight) were obtained from the Experimental Animal Center located in Kangwon National University (Korea). These animals were cared under conventional condition with suitable room temperature (about 23 °C) and relative humidity (about 60%). Constant dark/light cycle was controlled every 12 h, and freely accessible feed and water were provided to the animals. The protocol of this experiment was approved (approval no., KW-180124-1) by the Institutional Animal Care and Use Committee (IACUC) located in Kangwon National University. This protocol adhered to guidelines described in the current international laws and policies in “Guide for the Care and Use of Laboratory Animals” [43]. According to U.S. Department of Agriculture (USDA) pain classification (Policy ID No.: P209.03), a gerbil model of 5-min transient forebrain ischemia is a pain level D in the ischemic surgery involving pain or distress. The humane end point was determined by reduction in feed intake or weight loss of more than 20% of normal weight and intended to be euthanized immediately according to the guidelines form Johns Hopkins University [18]. In the present study, no animals showed cachexia or sings of humane endpoints. 

### 4.3. Administration of CMR and/or EALP Extract

The animals were randomly divided into eight groups (*n* = 7 in each group): (1) and (2) were the vehicle/sham and ischemia group: each was treated with vehicle (sterilized normal saline; 0.85% *w/v* NaCl) and underwent sham and ischemia/reperfusion operation, (3) and (4) were the 50 mg/kg YES-10/sham and ischemia group: each was treated with 50 mg/kg YES-10 and given sham and ischemia/reperfusion operation, (5) and (6) were the 100 mg/kg YES-10/sham and ischemia group: each was treated with 100 mg/kg YES-10 and subjected to sham and ischemia/reperfusion operation and (7) and (8) were the 200 mg/kg YES-10/sham and ischemia group: each was treated with 200 mg/kg YES-10 and undergone sham and ischemia/reperfusion operation. The vehicle/sham group was designated as a control group.

Each extract was orally administrated by using a feeding needle once a day for 1 week because extracts from natural resources had been taken orally in traditional medicine. Data concerned with absorption and metabolism of these extracts had rarely been reported, and the period of the treatment (7 days) was chosen according to our published papers [5,24].

### 4.4. Ischemia/Reperfusion Operation

The surgical procedure for ischemia/reperfusion injury was done as previously described [44]. In short, the gerbils were anesthetized by using an inhaler with a mixture of 2.5% isoflurane (Hana Pharmaceutical Co., Ltd., Seoul, Korea) in 33% oxygen and 67% nitrous oxide [45,46,47]. The ventral side of the neck was incised at the midline, and right and left common carotid arteries (CCA) were exposed. These CCA were simultaneously ligated with non-traumatic aneurysm clips. These clips were removed for reperfusion 5 min after CCA ligation. The complete occlusion of the CCA or reperfusion was confirmed in the central arteries located in the retinae by using an ophthalmoscope (HEINE K180®, Heine Optotechnik, Herrsching, Germany). During the surgical procedure, body temperature was monitored to control normothermic condition (37 ± 0.5 °C) with a rectal temperature probe (TR-100; Fine Science Tools, Inc., Foster City, CA, USA). Sham operated animals were given the same surgical procedure excluding ligation of the CCA. After this surgery, the gerbils were kept in an incubator (23 °C and 60% of humidity).

It is well known that a gerbil model of 5-min transient forebrain ischemia shows dramatic pyramidal neuronal death/loss in the hippocampal CA1 field at 4–5 days after ischemia/reperfusion [3]. In this regard, by using the above-mentioned model, survival of CA1 pyramidal neurons has been considered as a yardstick of neuroprotection when materials to have neuroprotective potentialities against ischemia/reperfusion such as single compound drugs or extracts from natural resources were investigated [25]. In addition, we selected “5 days”, not “1, 3 or 4 days”, after ischemia/reperfusion to investigate neuronal death/loss and reactive gliosis.

### 4.5. Tissue Preparation for Histological Examination

The preparation of histological tissues was carried out according to our published method [21]. In short, 70 mg/kg pentobarbital sodium (JW Pharm. Co., Ltd., Seoul, Korea) was intraperitoneally injected to the animals for deep general anesthesia at 5 days after ischemia/reperfusion operation [48,49]. They were perfused (flow rate, 6 ml/min; total perfused volume, 60 mL) for brain fixation via the ascending aorta with 0.1 M phosphate-buffered saline (PBS, pH 7.4) followed by 4% paraformaldehyde (in 0.1 M PB, pH 7.4). Their brains were harvested after fixation and post-fixed in the same fixative at room temperature for 4 h. The post-fixed brains were infiltrated with 30% sucrose (in 0.1 M phosphate buffer, pH 7.4) at room temperature for one day. The cryoprotected brains were serially sectioned into 30-μm frontal sections in a cryostat (Leica, Nussloch, Germany).

### 4.6. CV Staining

CV staining was performed to examine the pattern of cellular distribution in the hippocampus at 5 days post-ischemia. In short, as we previously described [50], the brain sections were stained with a solution of 0.1% (*w/v*) CV acetate (Sigma-Aldrich, St. Louis, MO, USA). These stained sections were subsequently incubated in serial ethanol bath in order to dehydration. Finally, they were prepared for permanent slides according to general method.

### 4.7. F-J B Histofluorescence Staining

F-J B (a marker for cellular degeneration) histofluorescence staining was carried out to examine neuronal death in the hippocampus at 5 days post-ischemia. Briefly, as previously described [51], the brain sections were mounted onto gelatin-coated slides and immersed with a solution of 0.06% potassium permanganate. These sections, subsequently, were incubated in a solution of 0.0004% F-J B (Histochem, Jefferson, AR, USA). These stained sections were dehydrated in serial ethanol and covered with glasses according to general method.

### 4.8. Immunohistochemistry

Immunohistochemistry was done to examine damage of neurons and changes (gliosis) of microglia and astrocytes in the gerbil hippocampus at 5 days post-ischemia. In short, as previously described [5], the brain sections were exposed to a solution of 0.3% H_2_O_2_ (in 0.01 M PBS, pH 7.4) and immersed in a solution of 10% normal goat or horse serum (in 0.01 M PBS, pH 7.4). These sections were incubated in each solution of primary antibody - mouse anti-NeuN (1:800, Chemicon, Temecula, CA, USA) for investigating neuronal damage, mouse anti-GFAP (1:1000, Chemicon, Temecula, CA, USA) for examining astrocytes and rabbit anti-Iba-1 (1:800, Wako, Osaka, Japan) for analyzing microglia - at 4 °C for 12 h. These incubated sections were reacted in a solution of biotinylated horse anti-mouse IgG or goat anti-rabbit IgG (1:250, Vector, Torrance, CA, USA) as a secondary antibody and subsequently immersed in a solution of avidin-biotin complex (1:300, Vector, Torrance, CA, USA). Finally, these sections were visualized by reacting with a solution of 3, 3’-diaminobenzidine tetrahydrochloride (DAB) (Sigma, St. Louis, MO, USA), and these immunostained sections were prepared as permanent slides.

### 4.9. Data Analysis

NeuN immunoreactive cells as neurons and F-J B positive cells as dead cells were counted. In brief, according to our published method [52], NeuN immunoreactive neurons were examined with a light microscope (BX53, Olympus, Hamburg, Germany). F-J B positive cells were examined with an epifluorescent microscope (Carl Zeiss, Göttingen, Germany) with blue (450–490 nm) excitation light and a barrier filter. Both microscopes were equipped with a digital camera (DP72, Olympus, Hamburg, Germany) that was connected to a PC monitor. We obtained digital images of NeuN immunoreactive neurons or F-J B positive cells in the same areas of the stratum pyramidal in the hippocampus proper. These captured cells were counted by using an image analyzing software (Optimas 6.5, CyberMetrics, Scottsdale, AZ, USA).

Quantitative analysis of GFAP and Iba-1 immunoreactivity was performed as relative optical density (ROD). As previously described [5], in short, digital images of GFAP and Iba-1 immunoreactive structures were obtained from the same regions of the hippocampus proper with a light microscope (BX53, Olympus, Hamburg, Germany) equipped with a digital camera (DP72, Olympus, Hamburg, Germany). Each image was calibrated into an array of 512 × 512 pixels, and each immunoreactivity was measured by a 0 to 255 gray scale system. Namely, background density in each image was subtracted, and ROD was calibrated by using Adobe Photoshop (version 8.0) and NIH image 1.59 software. A ratio of ROD was calibrated as percentage with the vehicle-sham group considered as 100%.

### 4.10. Statistical Analysis

Data obtained in this study were presented as the mean ± SEM. The data were measured by two-way analysis of variance (ANOVA) with a post hoc Bonferroni’s multiple comparison test to show differences among all the experimental groups. *P* < 0.05 was considered as indicating statistically significant.
